# Sertoli cell-specific ablation of *miR-17-92* cluster significantly alters whole testis transcriptome without apparent phenotypic effects

**DOI:** 10.1371/journal.pone.0197685

**Published:** 2018-05-24

**Authors:** Alicia Hurtado, Francisca M. Real, Rogelio Palomino, Francisco David Carmona, Miguel Burgos, Rafael Jiménez, Francisco J. Barrionuevo

**Affiliations:** 1 Departamento de Genética, Universidad de Granada, Granada, Spain; 2 Instituto de Biotecnología, Centro de Investigación Biomédica, Universidad de Granada, Armilla, Granada, Spain; 3 Departamento de Bioquímica y Biología Molecular I, Universidad de Granada, Granada, Spain; 4 Instituto de Investigación Biosanitaria de Granada, Universidad de Granada,Centro de Investigación Biomédica,Armilla, Granada, Spain; Centre de Recherche en Cancerologie de Lyon, FRANCE

## Abstract

MicroRNAs are frequently organized into polycistronic clusters whose transcription is controlled by a single promoter. The *miR-17-92* cluster is expressed in most embryonic and postnatal organs. It is a potent oncogene associated to several types of cancer and it is involved in several important developmental processes. In the testis, expression of the *miR-17-92* cluster in the germ cells is necessary to maintain normal spermatogenesis. This cluster is also expressed in Sertoli cells (the somatic cells of the seminiferous tubules), which require miRNAs for correct cell development and survival. To study the possible role of *miR-17-92* in Sertoli cell development and function and, in order to overcome the postnatal lethality of *miR-17-92*^*-/*^ mice, we conditionally deleted it in embryonic Sertoli cells shortly after the sex determination stage using an *Amh-Cre* allele. Mutant mice developed apparently normal testes and were fertile, but their testis transcriptomes contained hundreds of moderately deregulated genes, indicating that testis homeostasis is tightly controlled in mammals and that *miR-17-92* expression in Sertoli cells contribute to maintain normal gene expression levels, but is unnecessary for testis development and function. Our results show that significant deregulation of hundreds of genes might have no functional consequences.

## Introduction

Sertoli cells (SCs) are the epithelial supporting cells within the seminiferous tubules of mammalian adult testes. Their main function is to sustain spermatogenesis by providing structural support, nursing and regulating the function of germ cells (GCs) via signaling molecules. They also produce the seminiferous fluid and form the blood-testis barrier (BTB), an inter-SC specialized junctional complex formed by cell adhesion molecules that defines a basal and an adluminal compartment into the seminiferous epithelium and serves as an immunological barrier that creates a unique micro-environment for GC development. In addition, SCs control spermatogonial self-renewal and survival, as well as phagocytose apoptotic spermatocytes and cell debris derived from spermiogenesis [1]. SCs play an essential role in testis determination and differentiation. The expression of the testis-determining gene *SRY* in embryonic SC precursors leads to SC differentiation and commits them to surround GCs and to form testis cords, the anlagen of the seminiferous tubules. During embryonic development, SC products are necessary for preventing GCs from meiosis entry, as well as for Leydig and peritubular myoid cell differentiation, and regression of the Müllerian ducts, the anlagens of female secondary sexual organs [2, 3].

Micro-RNAs (miRNAs) are a family of small non-coding RNAs (~22 nucleotides) that regulate gene function at the post-transcriptional level by either preventing protein translation and/or promoting mRNA degradation [4]. The *miR-17-92* cluster, also known as *Mirc1*, is a polycistronic miRNA gene encoding six members *(miR-17*, *miR-18a*, *miR-19a*, *miR-20a*, *miR-19b-1*, and *miR-92-1*) which are highly conserved in vertebrates and expressed in practically all tissues analyzed during embryonic and postnatal stages [5–7]. In human and mouse, two paralog clusters exist, *i*.*e*. the *miR-106a-363* cluster comprising 6 miRNAS (*miR-106a*, *miR-18b*, *miR-20b*, *miR-19b-2*, *miR-92a-2* and *miR-363*) and the *miR-106b-25* one with 3 members *(miR-106b*, *miR-93* and *miR-25*). The *miR-17-92* cluster is a potent oncogene and it has been associated to several types of both hematopoietic cancers such as B-cell lymphomas, B-cell chronic lymphocytic leukemia and T-cell lymphoma, and solid cancers including retinoblastoma, pancreatic cancer and breast cancer, among others [8]. The *miR-17-92* cluster plays also important roles during development. Hemizygous deletions of *MIR17HG*, the *miR-17-92* cluster host gene, have been associated to the Feingold syndrome, an autosomal dominant condition characterized by multiple skeletal abnormalities. Homozygous *miR-17-92* null mutant mice died perinatally due to lung defects and cardiac hypoplasia [5] and partially phenocopied some skeletal abnormalities of the Feingold syndrome [9]. This miRNA cluster was also shown to play a role in B- and T-cell development [5], neural stem cell differentiation [10], and orofacial clefting [11].

In the testis, several studies have shown that the members of the *miR-17-92* cluster are expressed in GCs and conditional inactivation of the cluster in either embryonic GCs or the whole adult testis resulted in spermatogenic defects [12, 13]. The SC-specific deletion of the RNase III enzyme *Dicer* in mice revealed that miRNAs also play an important role in the development and survival of SCs [14, 15]. Several studies have provided evidence that the members of the *miR-17-92* cluster are expressed in SCs. All the members of the cluster were cloned from purified P6 SCs [14], *in situ* hybridization with LNA (Locked Nucleic Acid) on adult testes showed *miR-17* and *miR-20a* expression in SCs [12], and ulterior analysis of the small RNA transcriptome of SCs purified from mice at postnatal day 6 revealed high levels of expression for *miR-19a* and *miR-19b*, intermediated levels for *miR-1*7 and *miR-20a* and low levels for *miR-18a* and *miR-92a* [16].

Overall, available data show that miRNAs are required for normal development of SCs and that *miR-17-92* expression in GCs plays a role in spermatogenesis maintenance, but nothing is currently known on the role of this miRNAs cluster in postnatal and adult SCs. In order to overcome the postnatal lethality of *miR-17-92* ^*-/-*^mice and to uncover novel functions for *miR-17-92* during SC development, we conditionally deleted the *miR-17-92* cluster in embryonic SCs shortly after the sex determination stage using an *Amh-Cre* allele.

## Material and methods

### Mice

To induce SC-specific deletion of *miR-17-92*, we mated mice with a floxed allele of *miR-17-92* [5] to mice harbouring the SC-expressed transgene Amh-Cre [17], obtained from the Jackson Laboratory (Bar Harbor, ME, USA; Stock No: 007915 and 008458, respectively). They were bred in our animal house facilities and the resulting double heterozygous offspring carrying the *Cre* allele was backcrossed to homozygous *miR-17-92*^*flox*^ mice to obtain *Amh-Cre;miR17-92*^*flox/flox*^ mice. To evaluate Cre-activity, *Amh-Cre* and *Amh-Cre;miR17-92*^*flox/flox*^ mice were bred with the Cre-reporter mouse line *R26R-LacZ* [18] (Jackson Laboratory, Bar Harbor, ME, USA; Stock No: 003309). Primers and PCR conditions for *miR-17-92*^*flox*^ *and* for *R26R-EYFP* and *Cre* have been previously described [5, 19]. Mice with genotype *miR-17-92*^*flox/flox*^ lacking the *Cre* allele were used as controls. All animal experiments in this study were approved by the University of Granada Ethics Committee for Animal Experimentation (exp. No: 2011–341), and were performed in accordance with the relevant guidelines and regulations dictated by this Committee.

### Histological and immunostaining methods

After dissection, gonads were weighted and prepared for standard histological methods, including fixation, sectioning and staining with haematoxylin and eosin. Single and double immunofluorescence were performed as previously described [20]. The antibodies used and the working dilutions are as follows: Laminin, Sigma L9393, 1:100; ACTA2, Sigma A2547, 1:200; Claudin11, Santa Cruz Biotechnology sc-25711, 1:100; DMC1, Santa Cruz Biotechnology sc-8973, 1:100; PCNA, Santa Cruz Biotechnology sc-56, 1:100; CYP14A1 (P450scc), Santa Cruz Biotechnology sc-18043, 1:100; SOX9, Merck Millipore AB5535, 1:500; WT1, DAKO M3561 (clone 6F-H2), 1:500. Photomicrographs were taken in a *Nikon Eclipse Ti* inverted fluorescence microscope equipped with a *Nikon digital sight DS-U3* camera at a resolution of 2560 x 1920 pixels, using the *Nis-Elements-AR version 4*.*10*.*03*.*64bit*. Some immunofluorescence pictures were edited by changing the color levels tool of the *Gimp* image editor software to enhance contrast and minimize unspecific background fluorescence.

### Analysis of apoptosis

Apoptotic cells were labelled using the Roche TUNEL kit (Fluorescent In Situ Cell Death Detection Kit) according to the manufacturer’s instructions.

### Analysis of BTB permeability

An *in vivo* test was performed to study the permeability of the BTB in the testes of control and mutant mice. We used a biotin-labelled tracer compound (EZ-Link Sulfo-NHS-LC-Biotin tracer, Thermo Scientific) as described [20].

### Quantitative RT-PCR

Total RNA from E15.5 testes was isolated using the RNeasy Micro kit (Qiagen) including DNase I treatment. After checking RNA integrity, synthesis of cDNA was performed using Superscript II reverse transcriptase (Invitrogen). PCR was performed using the NZY qPCR Green Master Mix (Nzytech). Efficiency of the primers was tested by a standard curve assay, and only primers with amplification efficiency close to 100% were used. Each sample was measured in triplicates in two independent experiments. Ct values of samples were normalized to the corresponding Ct values of *Gapdh*, and relative expression levels were calculated by the ΔΔCt method [21]. Primer pairs used were 5’-GGTGGGGATTGTGACCAG-3’ and 5’-CGAGCAAACACGAAAATGAA-3’ for *miR-17-92hg*; and 5’-GATGACATCAAGAAGGTGGTG-3’ and 5’-TCATACCAGGAAATGAGCTTG-3’ for *Gapdh*.

### Transcriptome preparation

Total RNA was isolated from three *Amh-Cre;miR17-92*^*flox/flox*^ and three control (*miR17-92*^*flox/flox*^) testes at postnatal day 60 (P60) using the Qiagen RNeasy Midi kit following the manufacturer’s instructions. Subsequently, the six samples were sent to Macrogen Inc. (Seoul, South Korea). After passing a quality check, libraries were prepared using TrueSeq RNA Sample Prep Kit V2 and they were paired-end sequenced separately in an Illumina HiSeq 4000 platform, generating between 120 and 135 million reads per sample.

### Bioinformatics

The RNA-seq reads were mapped to the UCSC mm10 mouse genome using the STAR RNA-seq aligner (version 2.5) [22], and subsequently they were counted with the featureCounts function from the R subread package [23]. Only genes with 5 or more RPKM (reads per kilobase per million) in at least two of the samples were considered to be expressed and were used for further analysis. Analysis of differential gene expression was performed with edgeR [24].

Gene Ontology analysis was performed for differentially expressed genes (FDR < 0.01) using the Database for Annotation, Visualization and Integrated Discovery (DAVID) [25]. All the expressed genes were used as background.

To generate a circos plot, raw data from the previously transcriptomes reported by Soumillon et al. [26] where downloaded from the Gene Expression Omnibus (Acc. GSE43717) and processed with the same pipeline as our own data. We performed pairwise comparisons of the transcriptomes of the different cell types with the edgeR package [24, 27] from bioconductor and used the glmQLFTest() function to Conduct gene wise statistical tests. We then constructed lists of genes for each cell type. Genes more expressed in a cell type than in any of the others were included in the list of this particular cell type. After the genes were distributed among the different cell types we calculated the intersection of each gene set with the differentially expressed genes in our *SC-miR-17-92* KO testis. We then constructed tables as input data for the biocircos.js library [28] including the distribution of all differentially expressed genes in our *SC-miR-17-92* KO testis among the different cell types, their relative expression levels in log_2_FC and the predicted target sites for the four miR-17-92 seed families obtained from TargetScan (version 7.1, http://www.targetscan.org). The tables where exported from R in json format and included in javascript scripts that used the biocircos classes to draw the circos plot.

The transcriptome datasets generated during the current study are available in the Arrayexpress repository **E-MTAB-5914**. All bioinformatic data generated and analysed during this study are included in this published article (and its Supporting Information files).

## Results

### Generation of a Sertoli cell-specific conditional *miR-17-92* mutant mouse line

**Fig 1:**
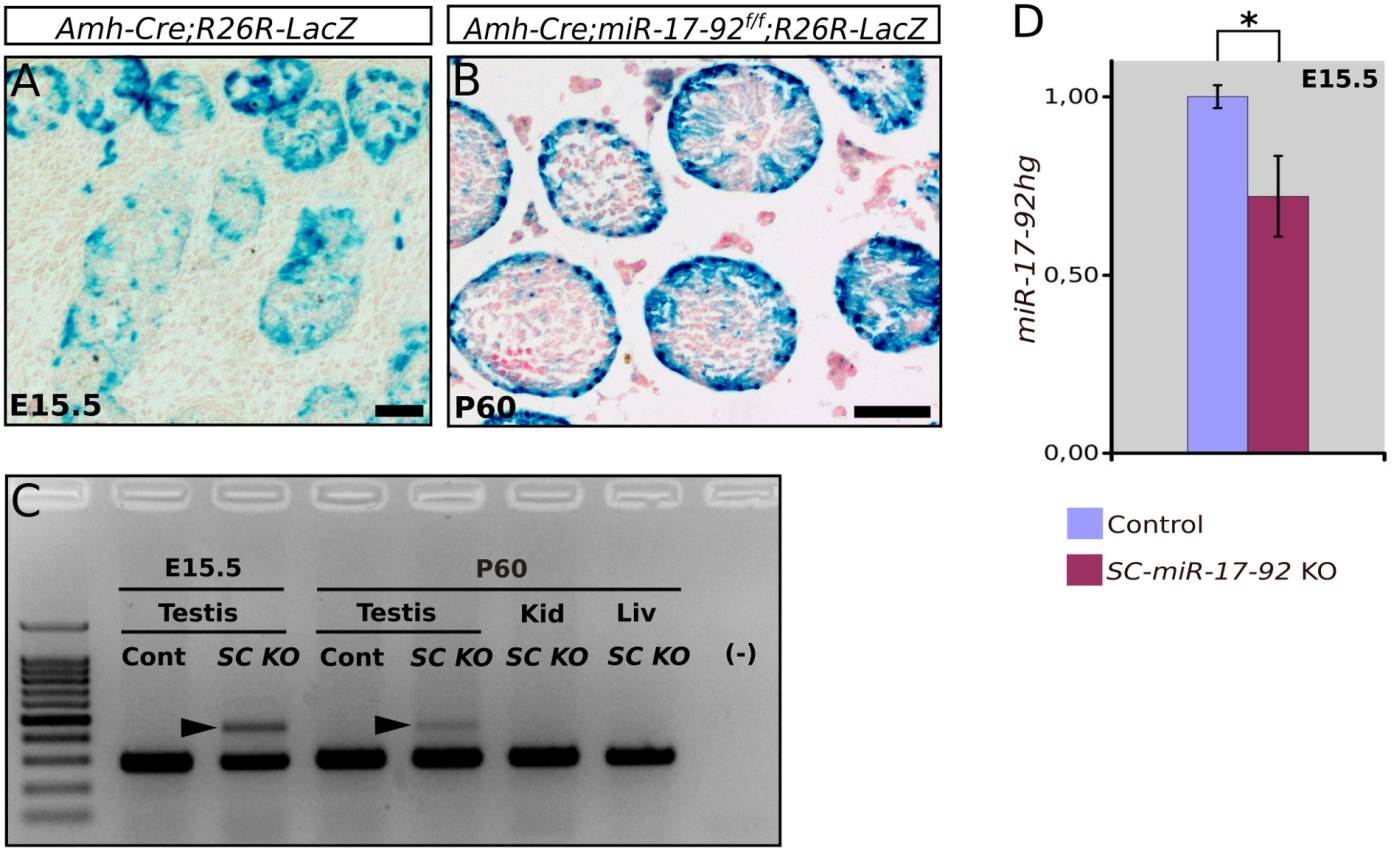
Confirmation that Sertoli cell-specific conditional *miR-17-92* mutant mice were generated. β-galactosidase staining of testes shows Cre-mediated recombination of the *R26R-LacZ* allele in Sertoli cells at both embryonic E15.5 (**A**) and P60 (**B**). (**C**) PCR genotyping shows that the 441 bp band specific for the deleted *miR-17-92*^*flox*^ allele (arrowheads) appears in testes of *SC-KO* mice, but not in those of control males or in other tissues (kid, kidney; liv, liver). (**D**) Quantitative RT-PCR for the detection of *miR-17-92hg* transcript levels in E15.5 control and *SC-miR-17-92* KO testes. Scale bars represent 20μm in A and 100μm in B.

To confirm SC-specific deletion of the *Amh-Cre* transgene, we generated mice carrying both the *R26R-LacZ* allele and the *Amh-Cre* allele. Subsequent β-galactosidase staining of E15.5 testes revealed Cre-mediated recombination of the *R26R-LacZ* allele exclusively in SCs (Fig 1A). Similar results were obtained in *Amh-Cre;miR-17-92*^*floxflox*^;*R26R-LacZ* mutants at 2 months (postnatal day 60, P60) (Fig 1B). Consistent with these observations, we detected a 441 bp PCR product specific for the deleted *miR-17-92*^*flox*^ allele in testes of *Amh-Cre;miR-17-92*^*flox/flox*^ mice (hereafter designated *SC-miR-17-92* KO), but neither in control testes nor in other *SC-miR-17-92* KO adult tissues (Fig 1C). Accordingly, quantitative RT-PCR of E15.5 control and *SC-miR-17-92* KO testes showed a significant reduction of about 30% in the transcript levels of the *miR-17-92* host gene (*miR-17-92hg*) in the mutant testes (n = 3; p = 0.041, two tail *t*-test; Fig 1D)

### Both somatic and germ cells are apparently normal in *SC-miR-17-92* KO testis

**Fig 2:**
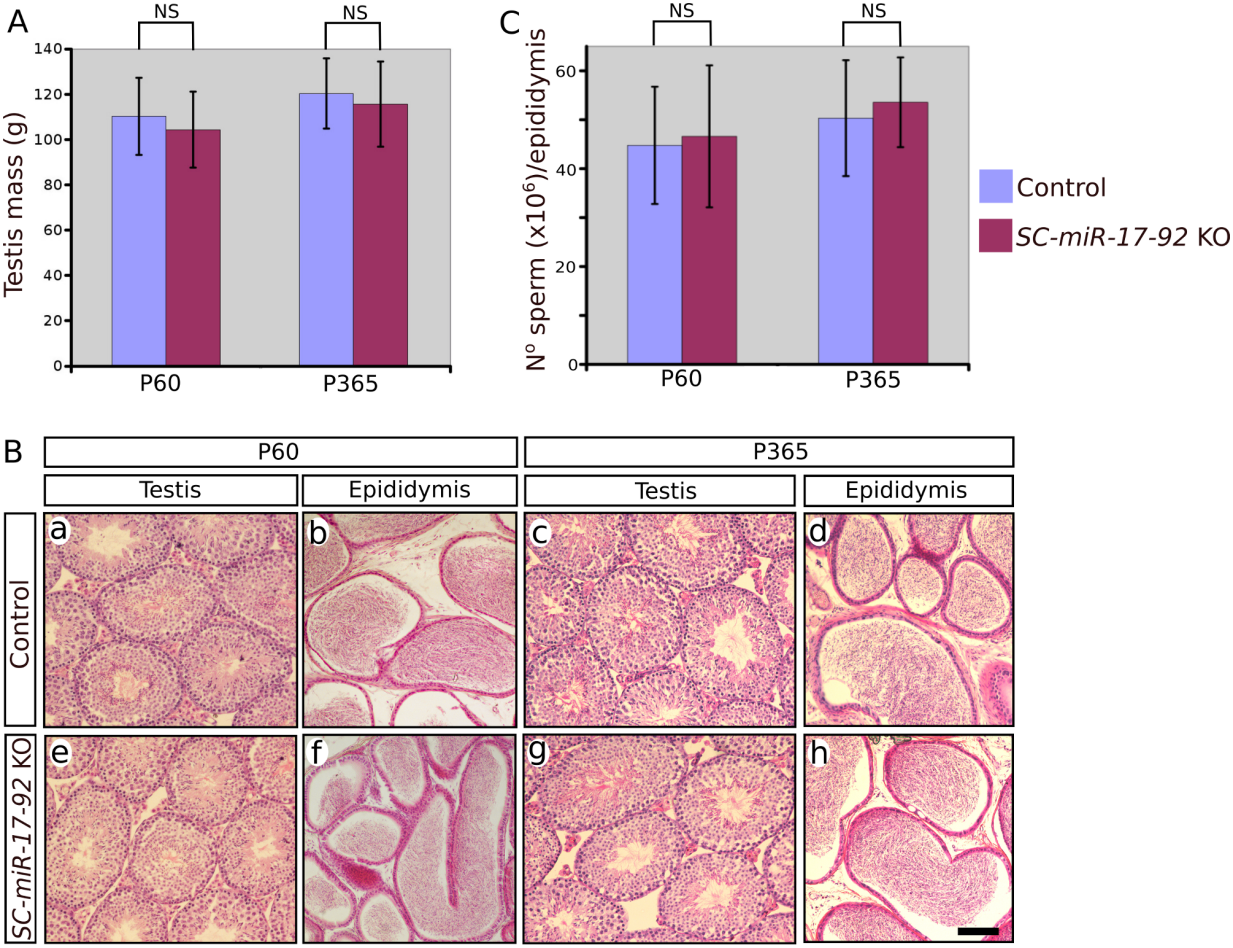
Testicular phenotype of *SC-miR-17-92* KO males at P60 and P365. Testis mass (**A**), histological features (**B**), and sperm counts (**C**) were similar in both mutant and control males. Scale bar shown in B represent 100μm for all pictures in B.

**Fig 3:**
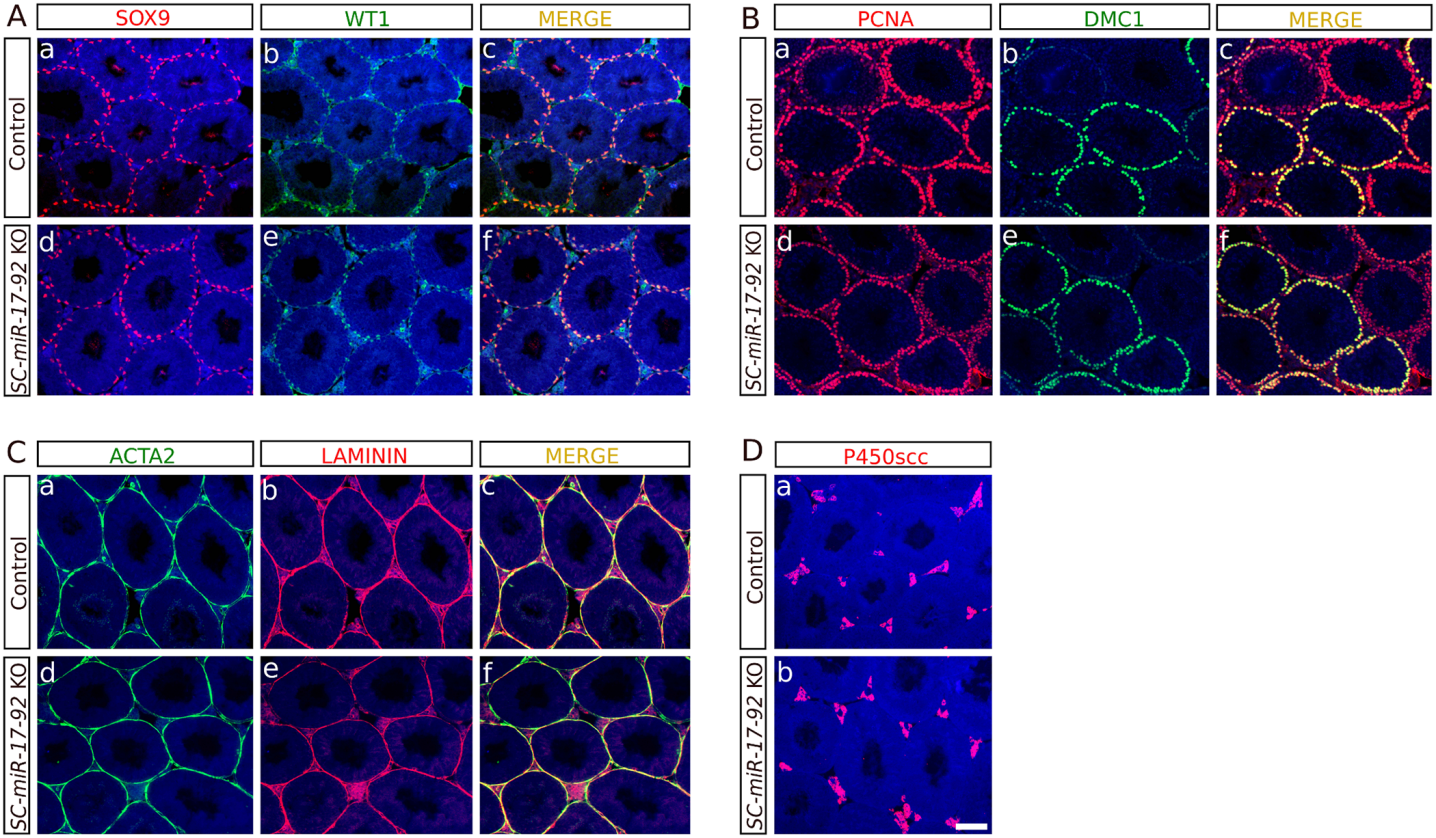
Unaltered expression of both somatic and germ cell markers in *SC-miR-17-92* KO testes. No difference between mutant and control testes was observed in the expression pattern of protein markers specific for Sertoli cells (SOX9 and WT1; A **a-f**), germ cells (PCNA and DMC1; **B a-f**), peritubular mioid cells (ACTA2; **C a** and **d**) and seminiferous tubule basement membrane (LAMININ; **C b** and **e; C c** and **f** show merged images), and Leydig cells (P450scc; **D a** and **b**). Scale bar shown in Db represent 100μm for all pictures in Fig. 3.

*SC-miR-17-92* KO mice were fertile and provided litters of apparently similar size than controls. At P60 we found no statistically significant difference between the testis mass of *SC-miR-17-92* KO and controls (n = 8; control 110 ± 17 mg; mutant 104 ± 16 mg; p = 0.48, two tail *t*-test). Histologically, 2 months old mutant testes were similar to controls showing seminiferous tubules of similar size and completing the spermatogenic cycle (Fig 2Ba and 2Be). Consistently, the epididymal tube was full of spermatozoa and epididymal sperm counts showed no difference between both conditions (n = 5; control 44.7 x 10^6^ ± 11.9 x 10^6^; mutant 46.6 x 10^6^ ± 14.5 x 10^6^; p = 0.82, two tail *t*-test; Fig 2C). The same condition persisted in year old mutant mice: 1) they were fertile, 2) showed no difference in testis mass when compared to controls (n = 5; control 120 ± 16 mg; mutant 115 ± 19 mg; p = 0.69, two tail *t*-test; Fig 2A), 3) their testes showed normal spermatogenesis (Fig 2Bg), their epididymal tubes were full of spermatozoa (Fig 2Bh), and their epididymal sperm counts were similar to those of controls (n = 5; control 50.3 x 10^6^ ± 11.8 x 10^6^; mutant 53.5 x 10^6^ ± 0.91 x 10^6^; p = 0.62, two tail *t*-test; Fig 2C).

Next, we studied the expression of several somatic and GC markers by immunofluorescence. SOX9 and WT1, two transcription factors expressed in adult SCs, are necessary for testis function and maintenance [19, 29]. Both control and *SC*-*miR-17-92* mutant testes exhibited the same expression pattern for the two proteins in SCs (Fig 3A). PCNA is expressed in mitotic spermatogonia as well as in zygotene and early pachytene spermatocytes [30] and the meiotic recombination protein, DMC1, is expressed in early spermatocytes [31] (leptotene-pachytene). No difference between mutant and control testes was observed in the expression patterns of these two proteins at both P60 (Fig 3B) and P365 stages (S1 Fig). Also, Laminin, a component of the basement membrane [32], and α-smooth muscle actin (ACTA2), a marker of peritubular myoid cells and arterial muscle fibers, showed similar expression in the two study groups (Fig 3C). Likewise, the expression of P450scc, a steroidogenic cytochrome expressed in Leydig cells, was also similar in both *SC-miR-17-92* KO and control testes (Fig 3D).

**Fig 4:**
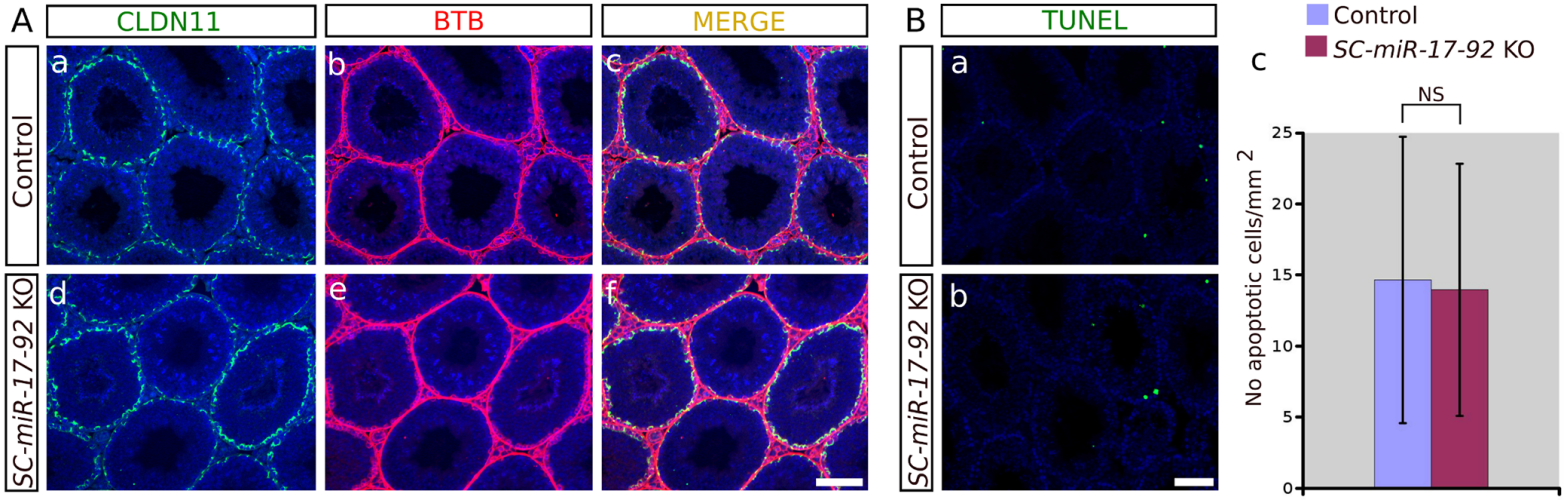
Analysis of the blood-testis barrier and apoptosis in *SC-miR-17-92* KO testes. (**A**) Both the expression pattern of CLAUDIN 11 (**a** and **d**) and the BTB function (**b** and **e**) are normal in mutant testes when compared to controls, (**c**) merged images a+b, (**f**) merged images d+e. (**B**) The incidence of apoptosis was similar in both mutant (**a**) and control (**b**) testes, as cell counts evidenced no significant differences between them (**c**). Scale bars shown in Af and Bb represent 100μm in A and B, respectively.

In addition, Claudin 11 (*Cldn11*), a major component of the tight junctions forming the BTB, did not show a different pattern of expression between the control and mutant condition (Fig 4Aa and 4Ad). Consistently, injection of a biotin tracer showed that the BTB in mutant males was impermeable, as observed in control testes (Fig 4Ab and 4Ae). Finally, TUNEL assay showed no increase in the frequency of apoptotic cells in *SC-miR-17-92* KO testes compared to control ones (n = 3; control: 14.65 ± 10.07 apoptotic cells/mm^2^; mutant: 13.96 ± 8.87 apoptotic cells/mm^2^; p = 0.23, two tail *t*-test; Fig 4Ba–4Bc). Altogether, these results show that normal testis function is preserved after SC-specific ablation of *miR-17-92*.

### The testicular transcriptome is altered in Sertoli cell-*miR17-92* KO male mice

**Fig 5:**
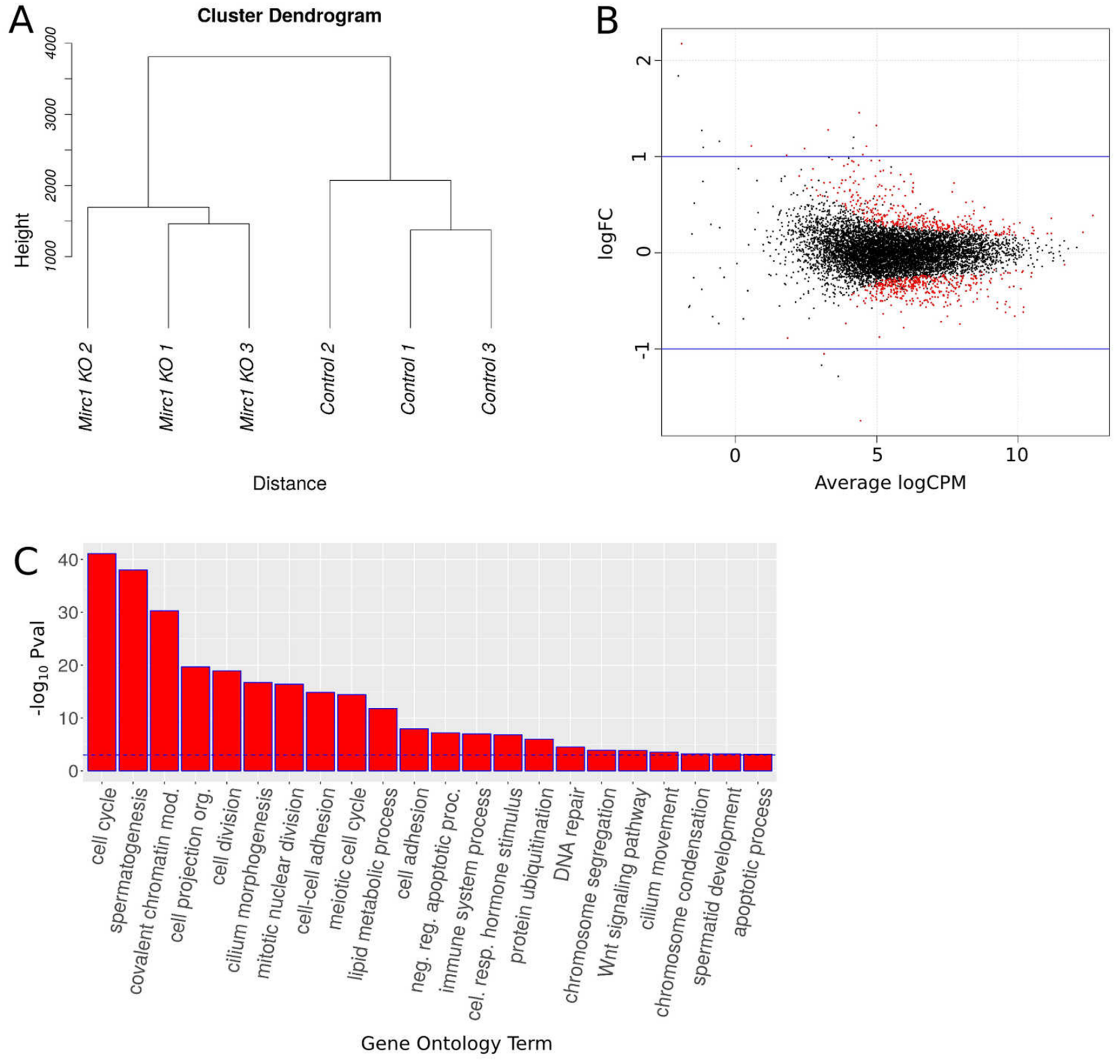
Transcriptome analysis of *SC-miR-17-92* KO testes. **A)** Hierarchical clustering analysis showed that the replicate testis samples of both *SC-miR-17-92* KO and controls grouped together. **B)** Smear plot of the differential expression test showed that the vast majority of the deregulated genes changed their expression levels by less than one log2 fold change (log2FC). **C)** GO analysis of *SC-miR-17-92* KO deregulated genes revealed a significant enrichment (P adj. < 0.05, blue dashed line) in terms associated to normal testicular functions.

We performed RNA-seq experiments on the adult testes of three *SC-miR-17-92* KO and three control males at postnatal day 60. The differences between the expression profiles of these samples were examined using a hierarchical clustering analysis (Fig 5A). Replicate samples from the same condition clustered together, indicating that the whole testis transcriptome is significantly and consistently altered when *miR-17-92* is deleted in SCs. To confirm this, we checked the presence of differentially expressed (DE) genes when comparing the two experimental conditions, and found 809 genes deregulated at P adjusted (FDR) < 0.01 (418 upregulated and 391 downregulated; see Table A in S1 File). Smear plot of the differential expression test showed that the vast majority of the DE genes were deregulated by less than 2 times, indicating that, despite a reproducible deregulation of many genes, the magnitude of the change was generally moderate (Fig 5B, see Table A in S1 File). Gene ontology (GO) analysis of DE genes revealed a significant enrichment (P adj. < 0.05) in terms related to normal testicular functions including spermatogenesis, meiotic cell cycle, chromosome segregation, cell-cell adhesion, cell-projection, and DNA-repair, among others (Fig 5C, see Table B in S1 File). Interestingly, although we deleted *miR-17-92* specifically in SCs, several GO terms referred to processes normally occurring in GCs (*e*.*g*. spermatid development and meiotic cell cycle), suggesting that spermatogenesis was also affected in the mutant mice. This observation is consistent with the well known notion that alteration of SC functionality is frequently associated to a failure in GC maturation [19, 33, 34]. Thus, we used the recently published transcriptomes of the five major testis cell populations (Sertoli, spermatogonia, spermatocyte, spermatid and spermatozoa) involved in spermatogenesis [26] to develop an algorithm to assign every differentially expressed genes in the *SC-miR-17-92* KO testes to particular cell types. First, we searched for differentially expressed genes performing pairwise comparisons of the transcriptomes of the cell types from the Soumillon’s et al. [26] study. Then we assigned the differentially expressed genes of our *SC-miR-17-92* KO to the specific cell type in which they showed the highest expression level in the Soumillon’s et al. [26] transcriptomes. We tested the genes assigned to particular cell types by looking for known specific gene markers of each cell type (see Tables C-G in S1 File). Consistently, major cell specific markers were assigned to the correct cell type: Sertoli (*Wt1*, *Cldn11*, *Cdh2*, *Sox8*, *Gata1*, *Gata4*), spermatogonia (*Gdnf*, *Cd9*, *Nanos2*, *Nanos3*, *Thy1*, *Bcl6*, *Gfra1*, *Epcam*) spermatocyte (*Sycp1*, *Sycp2*, *Sycp3*, *Rad51*, *Spo11*, *Syce1*, *Tex12*, *Eme1*), spermatids (*Prm1*, *Rsbn1*, *Rsb1nl*, *Tnp1*, *Tnp2*, *Theg*, *Nme8*, *Txnd8*), indicating that our approach clearly reflects cell-specific expression patterns.

**Fig 6:**
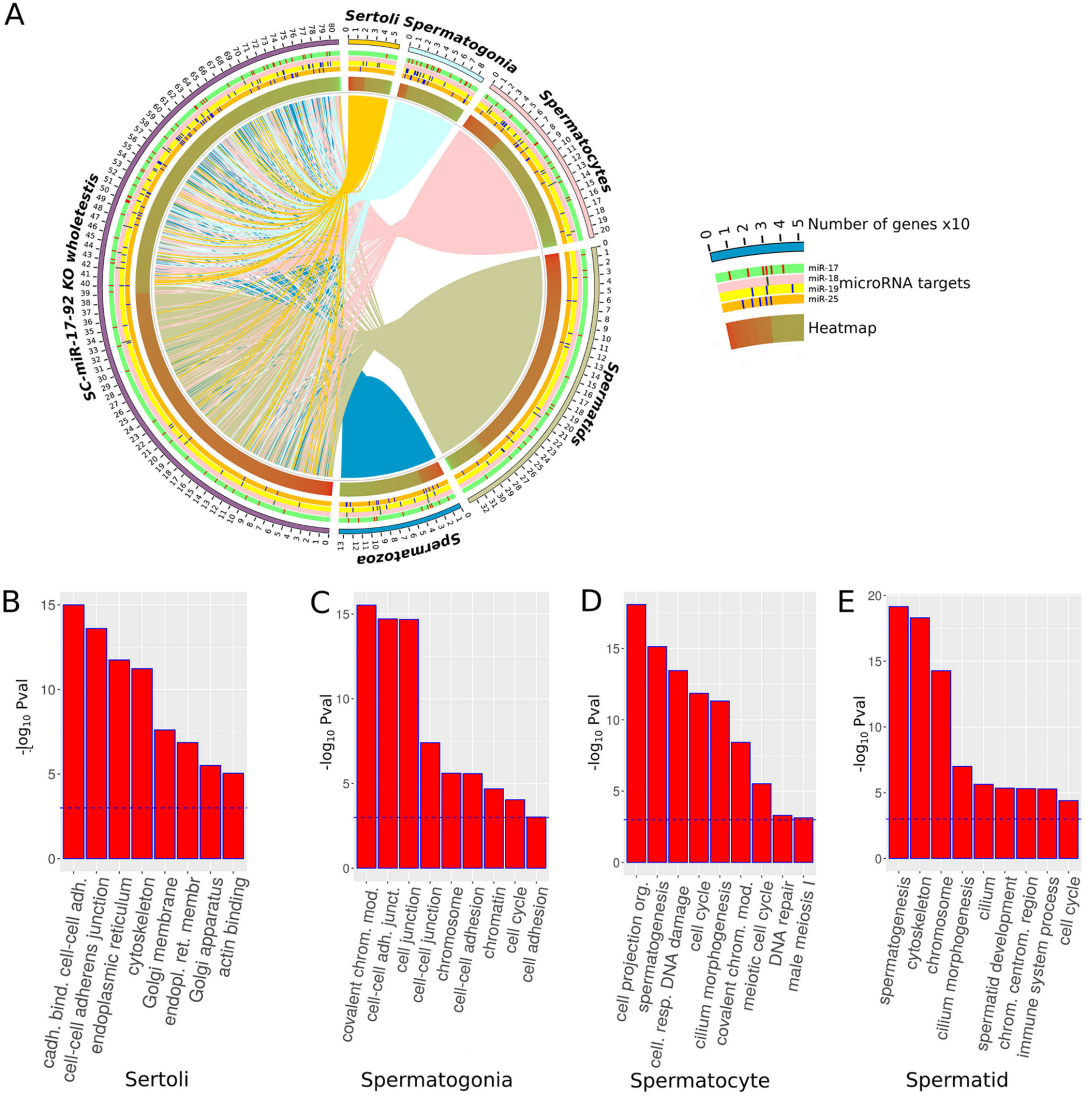
Assignation of differentially expressed genes to the five major cell types in the testis of *SC-miR-17-92* KO males. **A**) Circos plot showing the assignation to the different cell types of the differentially expressed genes in *SC-miR-17-92* KO testes. The color in the Heatmap track show relative expression level in *SC-miR-17-92* KO testes respect to control normal testes. Red indicate overregulated and green underregulated genes. **B-E**) Gene ontology enrichment analysis. Genes assigned to the different cell types appears to belong to GO terms normally related to the cell type to which they have been assigned according to the algorithm described. P adj. < 0.05, blue dashed line.

The circos plot in Fig 6A and Tables H-L in S1 File show the distribution of the DE genes in our *SC-miR-17-92* KO testes among the different cell types. It is noteworthy that both over- and under-expressed genes are not randomly distributed among the different cell types but most of the over-expressed genes belong to spermatids while most of the under-expressed genes belong to all other cell types, including SCs. In this cell type, we found 55 deregulated genes, 34 being downregulated and 21 upregulated (see Table H in S1 File). GO analysis of these genes revealed an overrepresentation in categories related to cell-cell adhesion and endoplasmatic reticulum (Fig 6B, see Table M in S1 File). Likewise, GO analysis of the deregulated genes in the other cell types showed enrichment in terms related to 1) cell junctions and cell cycle for spermatids, 2) meiotic cell cycle, covalent modification of chromatin and DNA repair for spermatocytes, and 3) cilium morphogenesis and spermatid development for spermatids (Fig 6C–6E, see Tables N-Q in S1 File), all of them referring to processes normally occurring in these cell types during spermatogenesis and spermiogenesis.

The individual miRNAs included in the *miR-17-92* cluster can be grouped into 4 families based on their seed sequences, which are the most important regions for target selection. Therefore, we searched for target genes that were deregulated in our mutant testis transcriptome, and found a number of target genes for each seed family (see Tables R-U in S1 File). However, they were not enriched in upregulated genes and were not preferentially located in the SC-specific list of deregulated genes (Fig 6A).

## Discussion

SC-specific ablation of *Dicer* resulted in abnormal testis development and spermatogenic failure, evidencing the importance of miRNAs in the differentiation and function of this cell type [14, 15]. Several authors have reported SC-specific expression of a number of miRNAs [14, 16] but little is known about the role of these translational regulators in SCs. Prompted by several studies showing the expression of members of the *mir-17-92* cluster in SCs [12, 14, 16, 35], and knowing their relevant roles in other organs and disease conditions (see the Introduction section), we decided to induce the conditional ablation of this miRNA cluster in embryonic SCs shortly after testis differentiation. Although the *SC-miR-17-92* KO adult testes showed apparently normal development and function, RNA-seq analyses evidenced consistent deregulation of many testicular genes in comparison to control testes, indicating that expression of the *mir-17-92* cluster in SCs is necessary to maintain the gene expression levels of the whole testis within normal values. Thus, the question arises as to why such an alteration of the transcriptome homeostasis does not result in an observable phenotype. In this regard, it should be noted that, despite the high number of deregulated genes in *SC-miR17-92* KO testes, the magnitude of the changes in their expression levels was generally modest (less than two times in most cases). Such a scenario, implying many deregulated genes showing just moderate changes in their expression levels, has been previously described for *miR-17-92*, and it is consistent with the proposed notion that its members act as fine-tuners of large gene networks rather than as major genetic switches of specific pathways [36]. The phenotypic consequences of transcriptome alterations induced by the absence of *miR-17-92* may be variable and difficult to predict. As an example, despite the fact that the number of deregulated genes in the embryonic tail buds of mice harboring a homozygous deletion for the *miR-17* seed family (~500) was lower than the observed in the same tissue of homozygous mutants for the *miR-19* seed family (~700), skeletal malformations were observed in the former mice (defects in axial patterning regulation), but not in the latter ones. Hence, it appears that the severity of the phenotype derived from the absence the *miR-17-92* cluster depends on the spatial and temporal cellular context. Most importantly, our result evidence that testis homeostasis must be strictly controlled as even when hundreds of genes were deregulated in all testicular cell types of *miR-17-92* KO testes, these showed no evident pathologic phenotype, conserving normal structure and function. Probably many other regulatory factors contribute to maintain testis homeostasis, as the simple ablation of *miR-17-92* is not enough to disturb it significantly. However, it is likely that mutant testes become more sensitive to stressful situations. Consistent with this idea, it was recently reported that hepatocyte-specific *miR-17-92*-deficient mice were fertile and apparently healthy, but their capacity of liver regeneration after tissue injury had resulted significantly reduced [37]. Hence, although no obvious testicular phenotype is observed, the transcriptome alteration we detected in our *miR-17-92* mutants might cause a suboptimal testis functional status.

Genes predicted to be miRNA targets become preferentially downregulated in cultured cells shortly after miRNA transfection [38]. Accordingly, transcriptome analysis of the embryonic tail buds of *miR-17-92-*deleted embryos at E9.5 (before the appearance of any developmental abnormalities) showed preferential upregulation of the predicted targets [36]. These observations contrast with our results, as we did not find a preferential upregulation of the predicted *miR-17-92* target genes in the transcriptomes of mutant testes. In our mutant mice, *miR-17-92* was depleted in SCs at the embryonic stage E14, but the testis transcriptomes were analysed nine weeks later, at the adult stage P60, as we wanted to see the effects in postnatal and adult testes. Hence, our transcriptomes do not reflect the primary effects of such a miRNA depletion, but probably many others induced subsequently by a chain reaction of deregulated genes that proceeded with time. The fact that we did not observe a severe or even moderate phenotype in the mutant testes, made it difficult to select a more convenient time point to analyze the transcriptome. Another limitation of our study is that we could only analyse the transcriptome of the whole testis, where spermatocytes and especially spermatids contribute very significantly to the total cell count and testis volume [26]. Hence, the search of miRNA targets using our transcriptome data is probably futile or even misleading.

GO analysis of deregulated genes in *SC-miR-17-92* KO testes showed a significant enrichment in biological processes associated with some GC-specific functions (*e*.*g*. spermatogenesis, meiosis, and spermatid development). As this alteration may not be a direct consequence of the misregulation of *miR17-92* target genes and their associated gene networks in SCs, we searched for SC-specific deregulated genes in *SC-miR-17-92* KO testes. GO analysis revealed that this SC-specific gene set was enriched in terms associated with cell adhesion, actin binding, and cytoskeleton. This is consistent with the fact that differentiating GCs progressively migrate across the seminiferous epithelium during spermatogenesis until they reach the tubular lumen. Throughout this migration, GCs remain attached to SCs via cell adhesion molecules, which prevent the sloughing of immature GCs from the seminiferous epithelium, a phenomenon that may lead to infertility [20, 39]. Moreover, specialized cell adhering structures established between adjacent SCs form the BTB, that isolates post-meiotic GCs from the systemic circulation [40]. In addition, SCs have a complex, well organized and functionally active cytoskeleton that requires abundant filament and microtubule proteins to maintain their structural integrity and hold neighbor germ cells [41]. For instance, *Cldn11*, which encodes Claudin 11, a principal component of the tight junctions forming the BTB, is one of the genes shown to be deregulated in our mutant mice. Mutation of this gene results in spermatogenic failure and male sterility [42]. Other affected genes in *SC-miR-17-92* KO testes were *Itgb1*, that encodes β1-integrin, an essential adhesion receptor necessary for homing the spermatogonial stem cells into the GC niche [43], *Tjp1* (or *ZO-1*) that encodes the tight junction protein 1, and *Jup* that encodes the junction plakoglobin. The two latter are essential adhesion proteins in SC-SC and SC-GC junctions [40] (see Tables M and N in S1 File). In the light of all these evidences, it seems plausible that SC-specific ablation of the *miR-17-92* cluster leads to the deregulation of the cell adhesion molecules present in the cell contacts by which the SC-GC cross-talk is established, thus resulting in an alteration of their transcriptomes.

In the mouse genome, two paralogues of the *miR-17-92* cluster exist, *i*.*e*. *miR-106b-25* and *miR-106a-363* and functional redundancy between the two first clusters has been revealed at the embryonic stage. On the other hand, expression of the third one has not been detected in most of the tissues analyzed to date including adult testes [5]. Individual members of the *miR-106b-25* cluster are known to be expressed in SCs [16], so they might compensate for the absence of the *miR17-92* cluster in the SCs of our mutant mice. To check this possibility, the testicular phenotype of *miR-17-92; miR-106b-25* double mutants must be analyzed, a work currently under way in our laboratory.

In summary, we generated conditional mutants in which SC-specific depletion of *miR-17-92* was induced during the embryonic development of the testis. Despite the fact that no phenotypic alteration was apparent in mutant adult testes, RNA-seq analysis revealed a consistent and reproducible alteration of their transcriptome, hundreds of genes being moderately deregulated. This indicates that testis homeostasis is tightly controlled in mammals and that the expression in SCs of the miRNAs included in the *miR-17-92* cluster contribute to maintain testis transcriptome within normal values, but are not essential to warrant normal testis development and function. While it is likely correct to consider that transcriptome profiles identify gene expression changes that do not necessarily link to the phenotype, this is usually not the case, as most studies report phenotypic defects in mutant animals. However, the reason is probably related to the rationale of most studies, in which profiling assays are performed once, and maybe because, a phenotype is observed, selecting the critical time point for “worst defect” or “putative defect of interest” in order to identify the candidate genes. Hence, our results evidence that significant deregulation of hundreds of genes, a situation frequently observed in many studies, not necessarily will have functionally relevant consequences.

## Supporting information

S1 Fig Expression of PCNA and DMC1 in control and SC-miR-17-92 KO testes at P365. No difference between mutant (d-f) and control (a-c) testes was observed in the expression pattern of PCNA (a,d) and DMC1 (b,e) at P365. Scale bar shown in f represents 100 μm for all pictures.
(TIF)

S1 File Bioinformatic analysis of the transcriptomes of control and *SC-miR-17-92* KO testes. (XLS)
