# Supplementary figures and images for: Sertoli cell-specific ablation of miR-17-92 cluster significantly alters whole testis transcriptome without apparent phenotypic effects
Source: PLoS One. 2018 May 24;13(5):e0197685. doi: 10.1371/journal.pone.0197685 (PMC5967698; doi:10.1371/journal.pone.0197685)

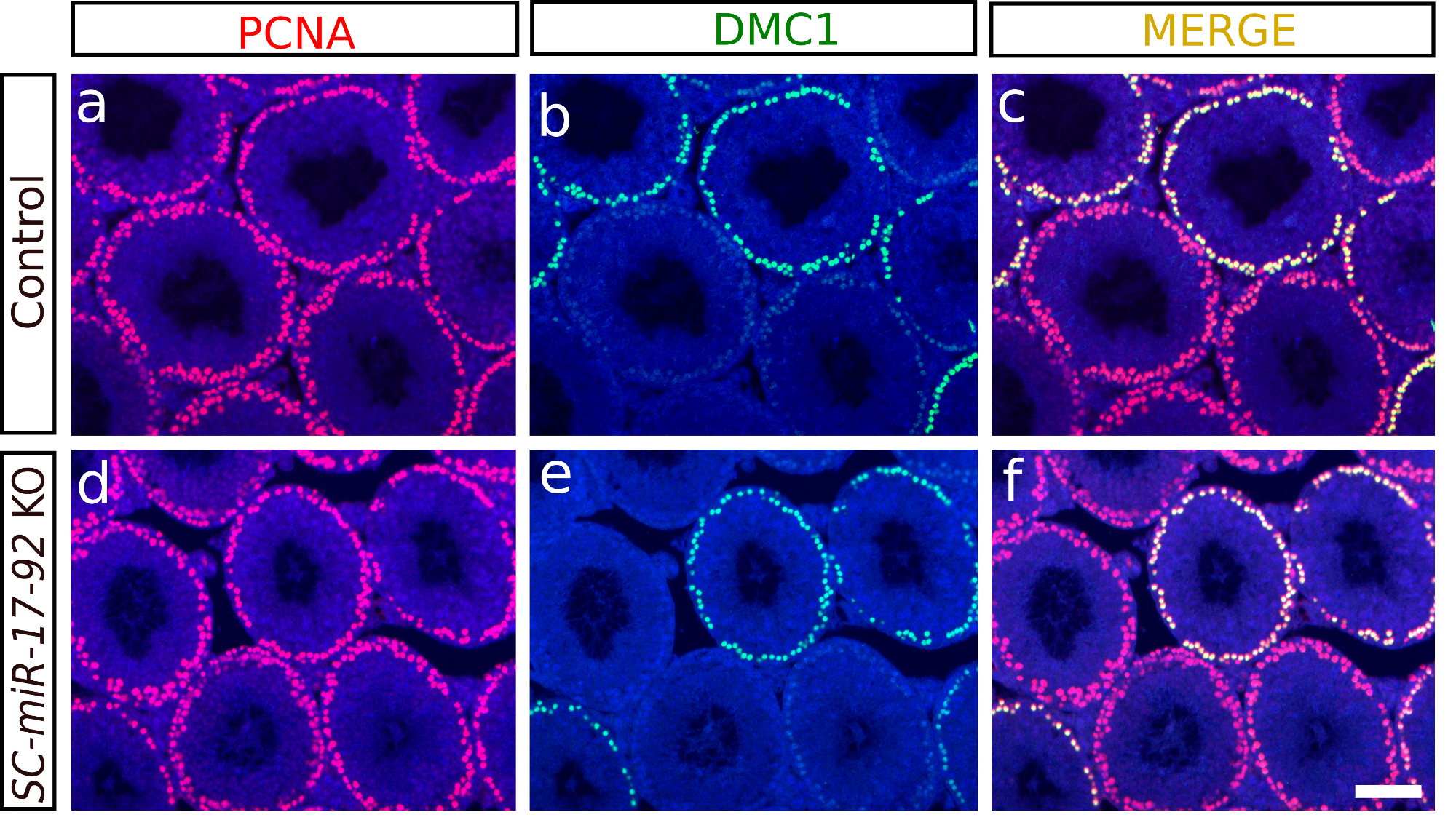

Supplement: S1 Fig — No difference between mutant (d-f) and control (a-c) testes was observed in the expression pattern of PCNA (a,d) and DMC1 (b,e) at P365. Scale bar shown in f represents 100 μm for all pictures. (TIF) [file pone.0197685.s001.tif]
